# Saliva specimen complements anal swab in assessing patients with COVID-19 for discharge from hospital

**DOI:** 10.1080/22221751.2021.1997339

**Published:** 2021-11-10

**Authors:** Chenghao Qiu, Zhigang Song, Jing Wang, Cheng Tian, Xingzhe Liu, Tingting Wu, Weisong Li, Shulin Zhang, Hongzhou Lu

**Affiliations:** Shanghai Public Health Clinical Center, Fudan University, Shanghai, People’s Republic of China

**Keywords:** COVID-19, discharge, clinical specimen, recurrent of viral detection, conversion days

## Abstract

Since December 2019, coronavirus disease 2019 (COVID-19) caused by SARS coronavirus 2 (SARS-CoV-2) has spread and threatens public health worldwide. The recurrence of SARS-CoV-2 RNA detection in patients after discharge from hospital signals a risk of transmission from such patients to the community and challenges the current discharge criteria of COVID-19 patients. A wide range of clinical specimens has been used to detect SARS-CoV-2. However, to date, a consensus has not been reached regarding the most appropriate specimens to use for viral RNA detection in assessing COVID-19 patients for discharge. An anal swab sample was proposed as the standard because of prolonged viral detection. In this retrospective longitudinal study of viral RNA detection in 60 confirmed COVID-19 patients, we used saliva, oropharyngeal/nasopharyngeal swab (O/N swab) and anal swab procedures from admission to discharge. The conversion times of saliva and anal swab were longer than that of O/N swab. The conversion time of hyper sensitive-CRP was the shortest and correlated with that of CT scanning and viral detection. Some patients were found to be RNA-positive in saliva while RNA-negative in anal swab while the reverse was true in some other patients, which indicated that false negatives were inevitable if only the anal swab is used for evaluating suitability for discharge. These results indicated that double-checking for viral RNA using multiple and diverse specimens was essential, and saliva could be a candidate to supplement anal swabs to reduce false-negative results and facilitate pandemic control.

## Introduction

Coronavirus disease 2019 (COVID-19) is a rapidly escalating pandemic that has spread to many parts of the world. Severe acute respiratory syndrome coronavirus 2 (SARS-CoV-2) was reported as the cause of COVID-19 by China in December 2019 [1,2]. As of 8 July 2021, about 185 million confirmed cases with more than 4 million deaths from more than 210 countries and territories were reported [3]. Although infections with SARS-CoV and the Middle East Respiratory Syndrome Coronavirus (MERS-CoV) have higher mortality rates than COVID-19, SARS-CoV-2 spreads much more rapidly and has a longer duration of RNA shedding than MERS-CoV and SARS-CoV [4–6]. Additionally, the recurrence of positive SARS CoV-2 PCR has been described in patients after they have been discharged from the hospital [7,8], which can further threaten pandemic control. Identifying and isolating infectious individuals by community testing and by testing clinically recovered patients before discharge from the hospital have been critical to contain the spread of the virus.

The reverse transcriptase polymerase chain reaction (RT-PCR) to detect SARS-CoV-2 is the primary method for the diagnosis of COVID-19 [9]. A range of clinical specimens has been reported to yield positive detection results, including oropharyngeal/nasopharyngeal swab (O/N swab), sputum, saliva, feces and anal swab, of which upper respiratory tract O/N swabs are the most common sample type in diagnosis [10]. However, growing evidence has revealed prolonged positive detection of nucleic acids in anal swabs and anal swab has been proposed as the optimal specimen type for evaluating COVID-19 patients for hospital discharge [11,12]. However, the duration of detected SARS-CoV-2 RNA shedding differs according to the different types of specimens [13]. For example, a study of 213 Chinese patients, including 205 throat swabs, 490 nasal swabs and 142 sputum samples, found false-negative rates of 40%, 27% and 11% for the throat, nasal and sputum samples, respectively [14]. Prolonged viral shedding accompanied by false-negative results was offered among the possible explanations for the recurrence of positive SARS CoV-2 PCR in patients discharged from hospital after two consecutive negative PCR results. Modified discharge standards, including optimal detection tests for viral RNA, are crucial to decrease the potential transmission revealed by the recurrence of positive PCR in COVID-19 patients.

There are few reports of the use of paired sampling to compare different specimen types to assess the duration of viral shedding. Here we compared saliva, O/N swabs and anal swabs in terms of assay sensitivity and number of days until assay negativity (conversion days) in a cohort of 60 confirmed imported COVID-19 patients. Daily samples were collected consecutively after the first sampling in each specimen type until the discharge of the patients. The resulting longitudinal study comparing specimen types for the detection of viral shedding can inform a revision of the hospital discharge standards applied to COVID-19 patients.

## Materials and methods

### Patients

Sixty COVID-19-confirmed patients who were imported into China and admitted into the Emergency Response Department of Shanghai Public Health Clinical Center (SPHCC) from 20 March to 20 April 2020 were included. Epidemiological history, clinical manifestations, laboratory test results and imaging test results were retrospectively collected from medical records. The study was approved by SPHCC Ethics Committee (Approval Number: YJ-2020-S046-02).

### Diagnosis and admission process

Individuals typically presented at Shanghai's district hospitals. Real-time reverse transcriptase polymerase chain reaction (RT-PCR) assays for confirming SARS-CoV-2 infection were conducted in local Centers for Disease Control and Prevention (CDC) according to WHO protocols [5]. Patients with positive nucleic acid test results were transferred by a negative-pressure ambulance to SPHCC for further treatment.

### Sampling during treatment and discharge standard criteria

Saliva, O/N swab and anal swab were collected from the patient every day during hospitalization, immersed in viral transport medium immediately and transferred to the lab in 30 min in a thermal box at 2–8°C. RT-PCR assays for confirming SARS-CoV-2 infection were conducted targeting ORF1ab/N gene by using kits from DAAN Gene CO. Ltd. (Catalog No.: DA0931) which have been approved by the National Medical Products Administration (NMPA). Patients were discharged when chest CT scanning showed inflammation absorption and the RT-PCR test was negative on 2 consecutive days in all three kinds of specimens.

### Sample collection

O/N swab and anal swab were collected following standard instructions for medical practice. Saliva samples were collected into 15 ml polypropylene centrifuge tube. Participants were instructed to repeatedly spit until approximately 2 ml of sample was obtained, thus avoiding mucous sections from oropharynx nor lower respiratory tract (i.e. sputum). Samples were immersed in viral transport medium immediately and transferred to the lab in 30 min in a thermal box at 2–8°C.

### Anti-COVID-19 IgM and IgG detection

Blood samples from the patients were collected and the serum samples were separated after centrifugation at 3000 rpm for 10 min. Qualitative testing for anti-COVID-19 IgM and IgG was performed by using two different in-vitro diagnostic products (IVD products) provided by Wondfo Biotech Co. Ltd.. Although the results of kit using Lateral Flow Method (Catalog No.: W195) were shown as “positive” or “negative” which couldn't supply the relative antibody level, the results of fluorescence immunochromatography assay (Catalog No.: Finecare™ 2019-nCoV Antibody Test) which provided relative light units (RLU) according to the level of antibody tested were applied to analyse the kinetics of IgM and IgG during infection.

### Statistical analysis

Enumeration data were expressed as percentages and rates. The Pearson correlation coefficient was used to measure the strength of conversion days of each two specimens. Differences in conversion days were compared by using Student's *t* tests. McNemar's test and Kappa test were applied to determine the significance of difference and concordance of positivity of viral RNA RT-PCR using different specimens separately. The level of significance was set at 0.05. SPSS 20.0 was used to analyse the data. All figures were created with Graphpad Prism7.

## Results

### Patients and samples

**Figure 1.**
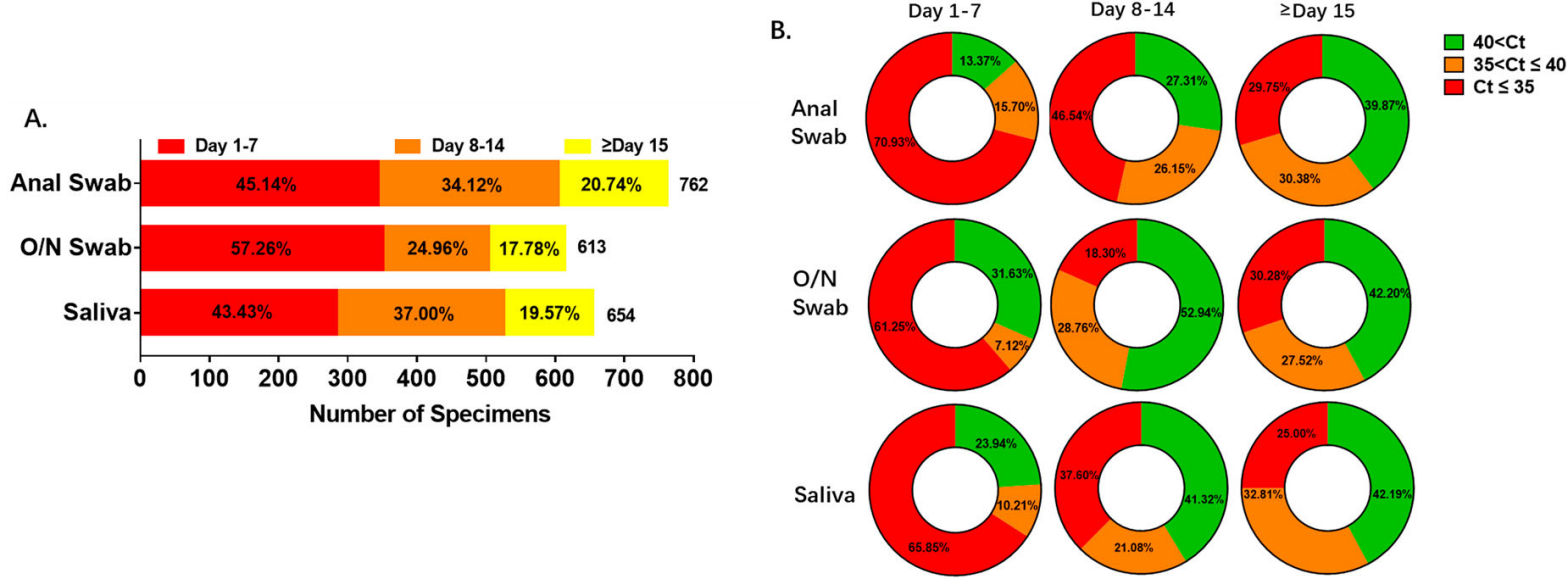
Sample profiles in each specimen type. Number of samples and viral RNA concentration are compared among the three specimen types. A. Total number of samples and proportion recorded in different periods after hospitalization. The percentage of samples collected during the first 7 days was higher in O/N swab than anal swab and saliva, while after the second 7 days the percentage was lower in O/N swab. B. Viral levels were compared among anal swab, O/N swab and saliva. Percentage of samples with higher viral level (Ct≤35), low viral level (35 < Ct≤40) and negative results (40 < Ct) are presented for the first 7 days, the second 7 days and the period after the second 7 days of hospitalization.

All the 60 enrolled patients (41 males and 19 females; Table 1) were imported from other countries and confirmed to be infected with SARS-CoV-2 by the CDC. During the treatment course in the hospital from 20 March to 12 May 2020, they provided three kinds of specimens: O/N swabs, saliva, anal swabs. Not all of the three kinds of specimens were collected from the first day of hospitalization, but sampling was applied every day once it began and continued until three consecutive negative results were detected after a positive result or until eight consecutive negative results were detected after the first sampling. In summary, 654 saliva samples, 613 O/N swabs and 762 anal swabs were tested (Figure 1A). Clinical records of the clinical examination, symptoms and viral RNA tests were retrospectively collected into the analyses. The severity of illness (mild, moderate, severe, critical) was evaluated according to the eighth edition of the Guideline for Diagnosis and Treatment of SARS-CoV-2 issued by the National Health Commission of the People's Republic of China [15]. Mild cases include mildly symptomatic with non-pneumonia and moderate cases include pneumonia. Severe disease refers to dyspnoea, respiratory rate ≥30/min, blood oxygen saturation ≤93%, partial pressure of arterial oxygen to fraction of inspired oxygen ratio <300 or lung infiltrates >50% within 24–48 h. 47 (78%) of the patients in this study were classified as moderate cases and the other 13 (22%) patients were mild cases (as recorded in Supplementary Table 2). The median age of the patients was 35 and ranged from 19 to 61 years old. Most of the patients (40%) were in the 30–39 years group (Table 1).

**Table 1.** Demographic characteristics of the patients.

Total	60
**Gender – no. (%)**	
Male	41 (68)
Female	19 (32)
**Age – no. (%)**	
10–19	4 (7)
20–29	12 (20)
30–39	24 (40)
40–49	14 (23)
50–59	5 (8)
60–69	1 (2)
Average	35.1 (range: 19–61)
Classification of clinical severity* – no.(%)	
Mild	47 (78)
Moderate	13 (22)

*Clinical severity of the symptoms was classified according to the eighth edition of the guideline for diagnosis and treatment of SARS-CoV-2 issued by the National Health Commission of the People's Republic of China.

### Sensitivity of viral RNA test with different specimens

**Figure 2.**
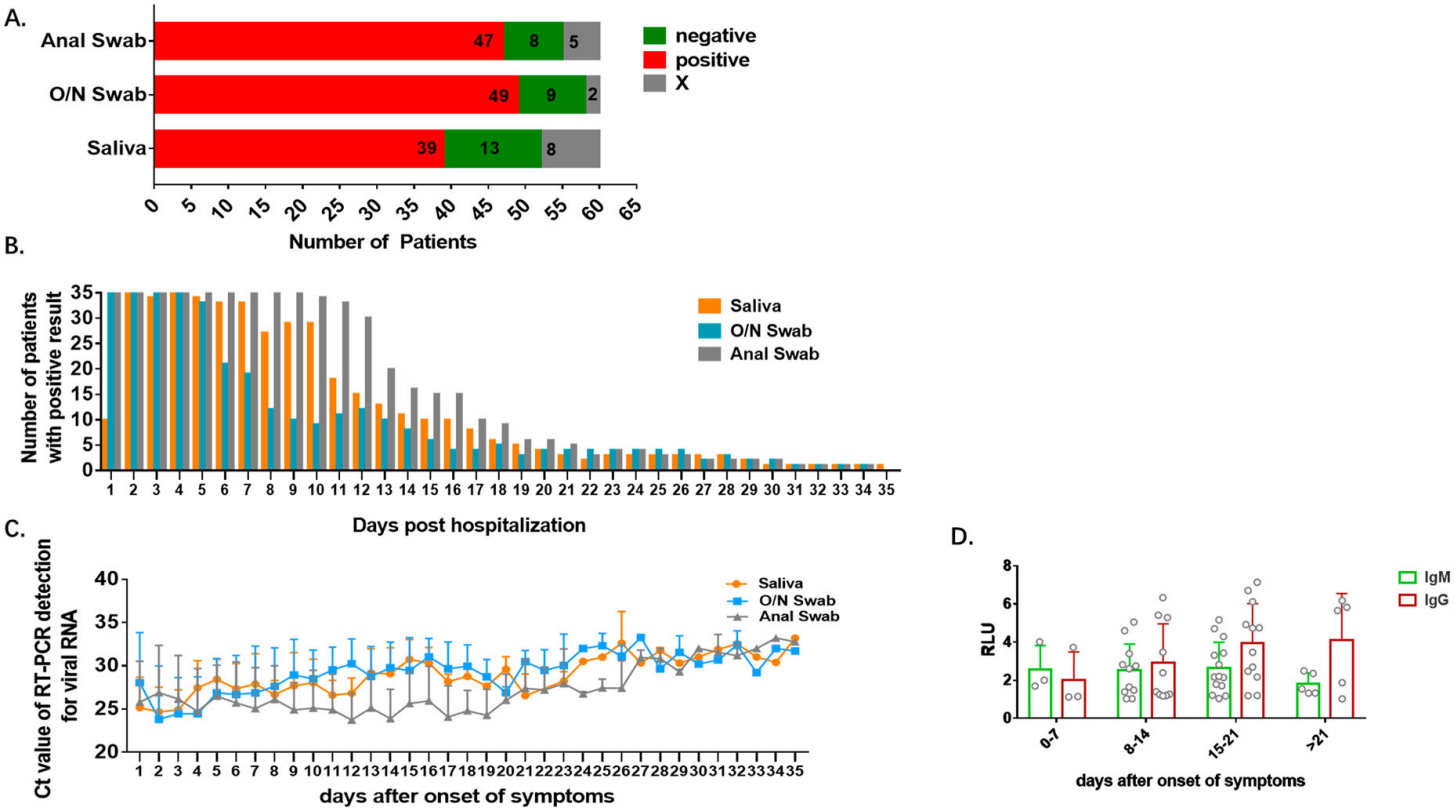
Detection profiles according to specimen type. Sensitivity and trend of viral RNA concentration are compared among anal swab, O/N swab and saliva. A. Number of patients detected (positive), undetected (negative) and with excluded results (X) in each specimen type. B. Number of patients with positive result in each specimen type each day after hospitalization. C. Viral RNA concentration in all detected patients by each specimen each day after the first positive result of each patient. D. The kinetics of anti-COVID-19 IgM and IgG in serum after onset of symptoms. RLU: the detected signals relative light units.

Patients with at least one confirmed positive result in any daily panel of three sample types were recorded as detected cases. Instances where a sample type was not collected during the first 7 days of hospitalization, and yielded no positive result after sampling began (marked X in the Supplementary Table 1), were excluded from the calculations of detection rates. The sensitivity provided by assay of a particular sample type was calculated as a percentage from the proportion of detected cases among the included cases (the 60 confirmed cases minus the excluded cases). Sensitivities of saliva, O/N swab and anal swab were 75% (39 detected cases/52 included cases), 84.48% (49/58) and 85.45% (47/55) respectively. 52 patients had at least one included result among the 3 specimen types (Figure 2A). 71.15% of the 52 patients were detected with all the three specimens (37/52) and 9.62% were negative with all the three specimens (5/52). Percentages of patients who were detected only with saliva, O/N swab or anal swab were 1.92% (1/52), 3.85% (2/52) and 3.85% (2/52) respectively. Percentages of patients who were negative only in saliva, O/N swab or anal swab were 7.69% (4/52), 1.92% (1/52) and 0 (0/52) as shown in Table 2. Results of McNemar's test revealed positivity of anal swab-based RT-PCR were not significantly different from that of saliva (*P* > 0.05) nor was O/N swab (*P* > 0.05). In addition, results of Kappa test suggested that the positivity of anal swab-based RT-PCR was statistically concordant with that of saliva (*P* < 0.001), which was the same with that of O/N swab (*P* < 0.001), and the magnitude of concordance was moderate with 0.4 < kappa value < 0.75 (Table 3).

**Table 2.** Profiles of RT-PCR results using different specimens.

Profiles of results			Number of patients	Percentage in total (%)
Saliva	Oropharyngeal swab	Anal swab		
+	+	+	37	71.15
+	–	–	1	1.92
+	+	–	0	0
+	–	+	1	1.92
–	+	+	4	7.69
–	+	–	2	3.85
–	–	+	2	3.85
–	–	–	5	9.62

**Table 3.** Difference and concordance of saliva and O/N swab with anal swab as reference.

		Anal swab		McNemar's test	Kappa test
		Positive	Negative		
**Saliva**	Positive	38	1	*P* = 0.125	Kappa value = 0.588
	Negative	6	7		*P* < 0.001

**O/N swab**	Positive	41	2	*P* = 0.999	Kappa value = 0.649
	Negative	3	6		*P* < 0.001

### Profiles of results from different specimens

Most of the specimens were collected during the first 7 days after admission. The number of samples decreased as the patients converted to assay-negativity. Only about 20 percent of samples were collected in the third week (Figure 1A). Viral RNA level was very high in samples collected during the first week and declined in the following weeks. This pattern was seen in all of the three sample types. As shown in Figure 1(B), 70.93% of anal swabs showed high viral RNA level and 15.70% were weakly positive in the initial 7 days. These percentages were higher than with the other two kinds of specimens. As the viral shedding declined in the second 7 days, differences in viral RNA level between the three samples became much greater. 72.69% of anal swabs were confirmed with viral RNA, while the percentage in saliva and O/N swabs were 58.68% and 47.06% respectively; 46.54% of anal swabs and 37.6% of saliva had high RNA level and these were much higher than in O/N swabs (18.3%). After the second week, the percentages of samples with high, medium and low viral RNA content were almost the same in the three sample types (Figure 1B).

### Conformity and differences between the results with the three sample types

As shown in Figure 2(A), overall, the saliva-based test gave a positive result 432 times out of the 654 tests completed (66.06% positivity), the O/N swab positivity rate was 61.17% (375/613), anal swab positivity was 76.38 (582/762). The number of patients with positive results was almost the same in the initial 5 days after hospitalization except for the first day in which 44 patients did not provide a saliva sample. The number of patients with positive result in O/N swabs declined from day 6 and was much lower than in saliva and anal swabs. The positivity rate of saliva declined from day 7, while that of anal swab declined from day 13, which suggested a prolonged viral RNA shedding in saliva and anal swab (Figure 2B). The level of viral RNA was very high in the initial samples. Kinetics of viral RNA level suggested higher viral level in anal swab than that in saliva and O/N swabs. Viral RNA abundance in saliva and O/N swabs declined gradually from day 7 after the onset of symptoms while that in anal swabs remained high till day 19 (Figure 2C). The level of COVID-19 specific IgM increased during the first 3 weeks after the onset of symptoms and went down after that, whereas IgG remained at high level after 3 weeks (Figure 2D).

**Figure 3.**
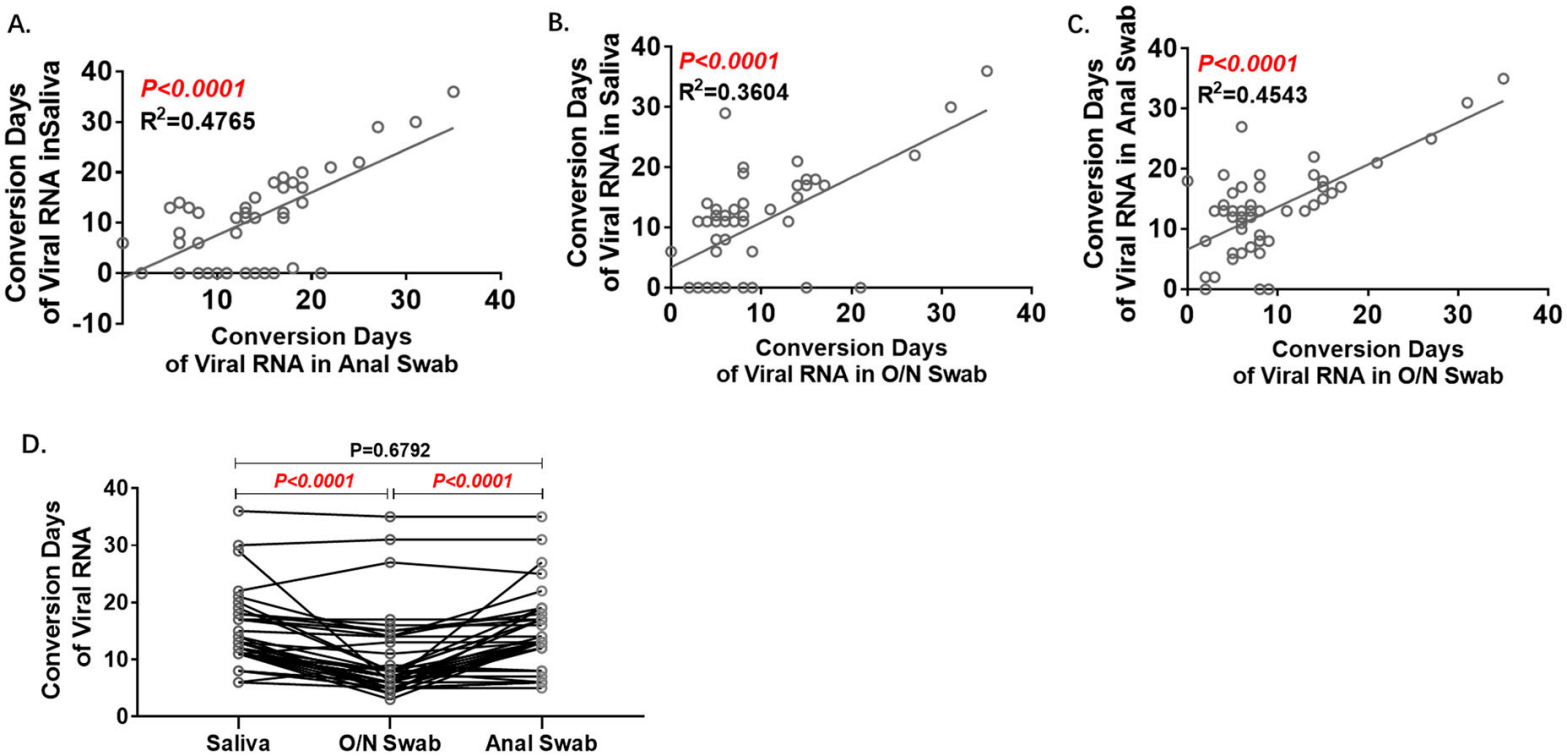
Analyses of Conversion Days comparing pairs of specimens. Conversion Days was calculated as days from hospitalization to the last positive viral RNA result. The Pearson correlation coefficient was used to measure the strength of the correlation between conversion days of each two specimens. Correlation of Conversion Days between anal swab and saliva(A), O/N swab and saliva (B), and O/N swab and anal swab (C). D. Comparison of Conversion Days between each pair of saliva, O/N swab and anal swab (Paired t test).

Conversion days were statistically correlated between any two of the three kinds of samples, and the conformity between saliva and anal swab results was better with lower R2 (Figure 3A–C). Among the 37 patients with positive results in all three sample types, the Conversion Days of the O/N swab were the shortest (Figure 3D).

**Figure 4.**
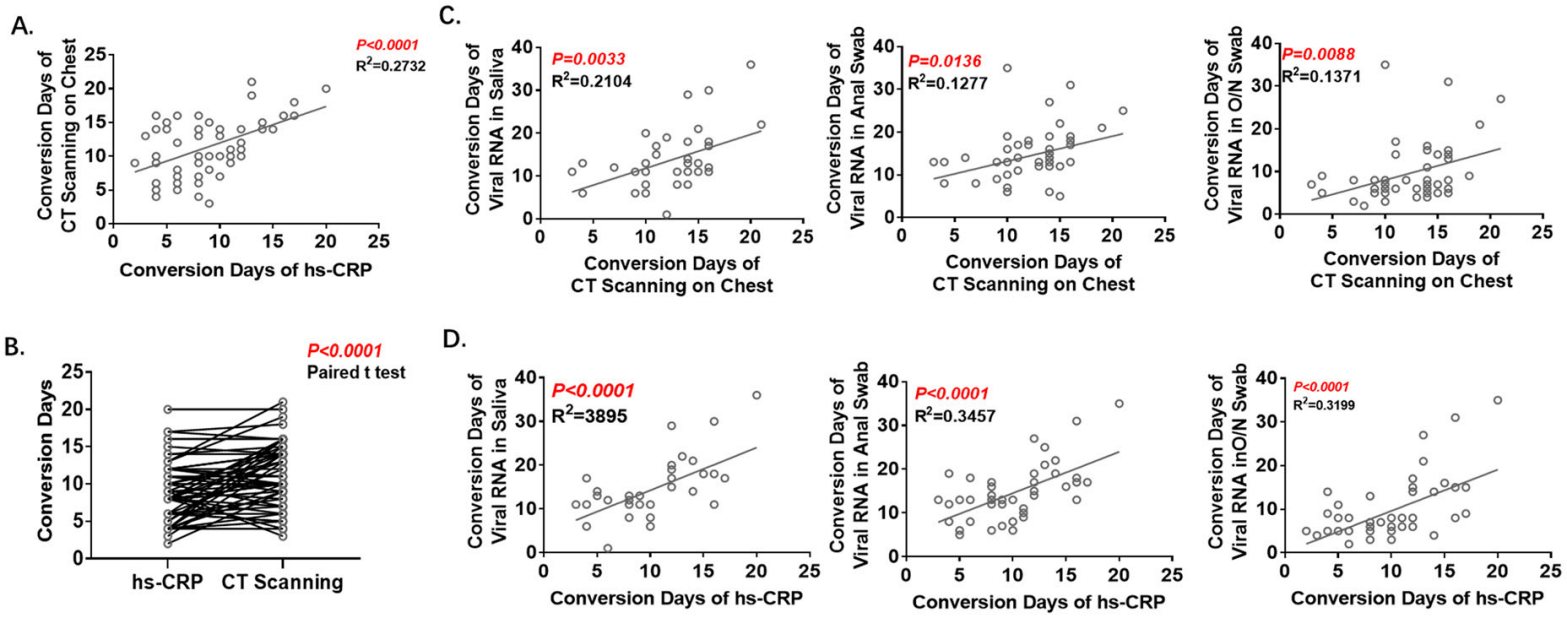
Analyses of Conversion Days of CT scanning and hs-CRP. A. Correlation of Conversion Days between CT scanning and hs-CRP. B. Comparison of Conversion Days between CT scanning and hs-CRP (Paired t test). C. Correlation of Conversion Days between CT scanning and RT-PCR with each of the three specimen types. D. Correlation of Conversion Days between hs-CRP and RT-PCR with each of the three specimen types. The Pearson correlation coefficient was used to measure the strength of the correlation.

CT scanning is regarded as providing one of the crucial criteria in the diagnosis of COVID-19, and hyper-sensitive-CRP (hs-CRP) is a sensitive indicator of the degree of inflammation. In this patient cohort, Conversion Days of hs-CRP and CT scanning were statistically correlated (Figure 4A) and Conversion Days of hs-CRP were much shorter (9.3 ± 0.5343 vs. 11.6 ± 0.5514; Figure 4B). Conversion days of all three tissue samples were correlated with those of CT scanning (Figure 4C) and hs-CRP (Figure 4D). The correlation with saliva was stronger than that with O/N swab or anal swab.

### Lack of association between clinical symptoms and conversion days

**Figure 5.**
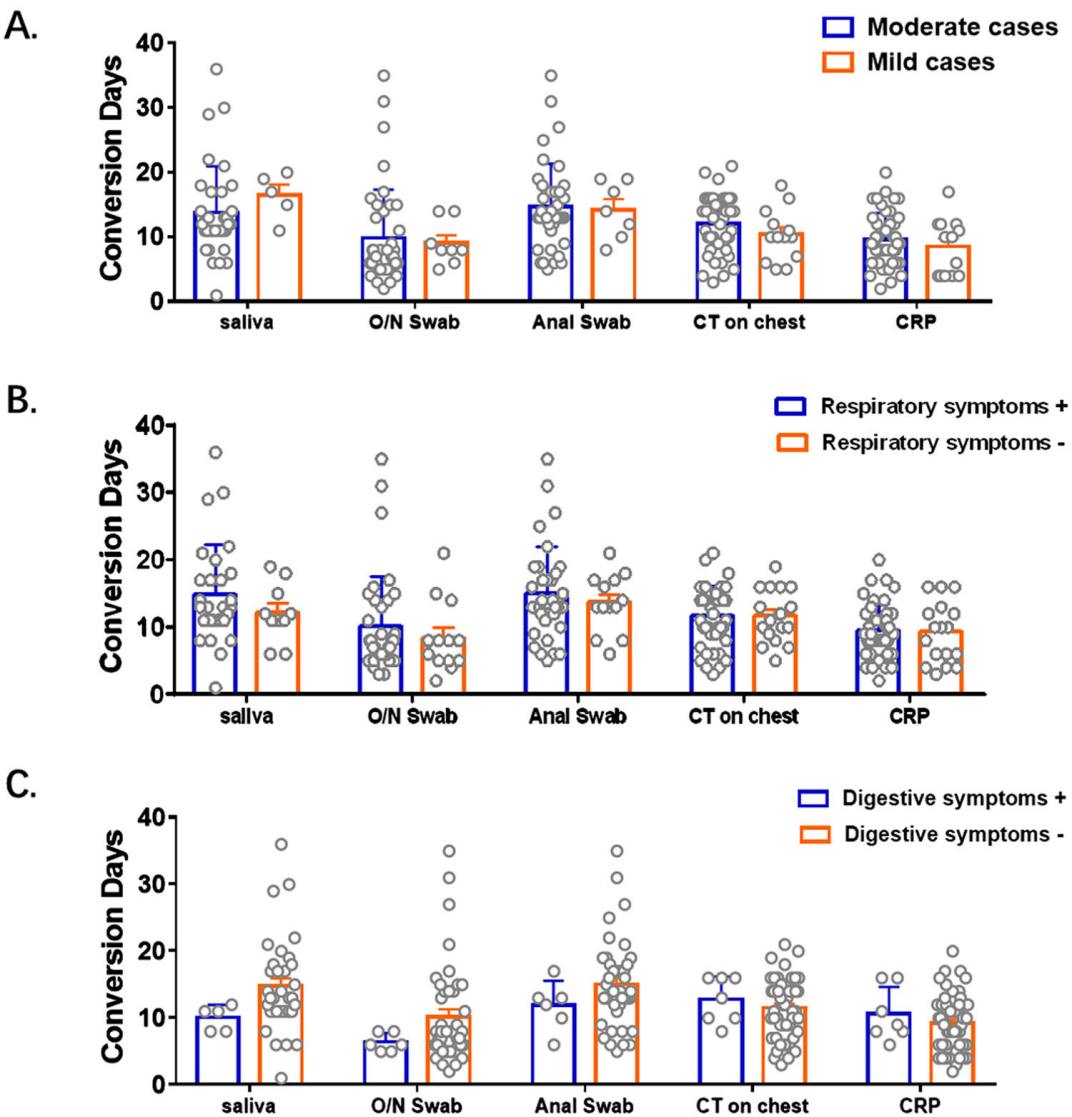
Impact of symptoms on conversion days of viral RNA and other clinical test. Patients were grouped according to their severity of symptoms (A), respiratory symptoms (B) and digestive symptoms (C) respectively. Conversion Days were compared. Difference between each two subgroup were determined by Student's t test.

Patients here were classified into mild to moderate cases of COVID-19, and some of them had symptoms of respiratory and/or digestive system disease. Viral load was directly linked with disease severity with higher viral loads leading to severe forms of the infection [16]. There have been many reports involving the impact of symptoms on viral RNA detection using various samples. Patients with respiratory symptoms were reported to have higher viral RNA levels in nasopharyngeal swabs than those without respiratory symptoms [17]. Similarly, viral load was higher in patients with digestive symptoms than in those without digestive symptoms [18]. Besides, conversion days were reported to be prolonged significantly in severe cases than mild cases [16]. However, researches on the impact of symptoms on conversion days were rare. In the cohort here, conversion days of viral RNA, CT scanning and CRP were comparable between patients with different severity of diseases, with/without respiratory symptoms and with/without digestive symptoms (Figure 5). Symptoms were suggested to have little impact on the conversion days of viral RNA in various specimens in mild-to-moderate COVID-19 cases.

## Discussion

According to the guidelines of the eighth edition of the Guideline for Diagnosis and Treatment of SARS-CoV-2 issued by the National Health Commission of the People's Republic of China [15], a patient can be discharged from hospital after two consecutive negative results of SARS-CoV-2 RT-PCR assay at least 24 h apart in a clinically recovered COVID-19 patient. Even though WHO removed the requirement of negative RT-PCR results in the latest recommendations for the discharge criteria issued on 27th May 2020, they also encouraged countries to continue testing patients, if they can do so, for systematic data collection that will enhance understanding and better guide decisions about infection prevention and control measures. The recurrence of SARS-CoV-2 after patients had been discharged from hospital with two consecutive negative results has been reported. The occurrence of false-negative assay results is one of the determinants of recurrence, which ranged from 2% to 29% according to a meta-analysis that included 957 hospitalized patients [19]. The cause of false negatives can be in the source of specimens, the sampling procedure, and the sensitivity and specificity of the test kit [7]. However, a contributory factor is that the course of SARS-CoV-2 infection remains unclear, notably in the duration of viral shedding in different specimens.

The cellular receptor for SARS-CoV-2, Angiotensin-converting enzyme 2 (ACE2) is most abundantly expressed on the surface of alveolar epithelial cells and small intestine epithelial cells [20,21]. Consequently, viral shedding may be through different routes during different phases of viral infection. In similarity to the situation for SARS-CoV and MERS-CoV patients, intestinal infection was reported to be detected in the later stage of infection, indicating that the clearance time of SARS-CoV-2 in the digestive tract was later than in the respiratory tract [22]. Prolonged SARS-CoV-2 detection in anal swab has been verified [6,12], which rendered anal swab as the proposed optimal specimen for determining hospital discharge and termination of compulsory isolation for COVID-19 patients.

In this study, we found that the duration of viral detection in saliva samples was similar to the duration in anal swab samples, without a statistical difference, and much longer than that in O/N swab samples. However, prolonged SARS-CoV-2 detection in saliva beyond that in anal swab was observed in 12/60 patients (20%; Table S2), which gives a cause for concern about using the anal swab as a solo standard for discharge. We propose a “double-check” using multiple specimens, especially including saliva, as the modified choice in assessing patients for discharge.

Saliva testing has been valued because it is suitable for self-collection, which can reduce the strain on the healthcare workers, for example in sample collection and consumption of personal protective equipment [23]. Despite this, controversy remains surrounding the sensitivity of tests of saliva compared with other specimens. Saliva is not a regular sample monitored in clinical treatment and follow-up. Unstandardized collection and processing are inevitable in self-collection, which could make the evaluation of sensitivity unreliable, but this can be corrected during inpatient sampling [24].

Most studies have found greater or similar sensitivities for saliva compared with O/N and anal swab-based tests. The sensitivity of saliva was underestimated in this study because of irregular sampling during the first week after admission. There were eight excluded patients in saliva sample data, those who did not provide saliva in the initial 7 days and were consistently negative of viral RNA thereafter. Viral loads are higher in the early days and in consequence false negatives were disproportionately increased in saliva sample data compared to O/N and anal swab sample data.

In addition to viral RNA detection, CT scanning is regarded as one of the standards for the diagnosis and discharge of patients [25,26]. We found that the mean Conversion Days of CT scanning was 12 days (range: 3–20 days), which was shorter than that of saliva (14.1 days, range: 6–36 days) and anal swab (14.6 days, range: 6–35 days), and longer than O/N swab (9.6 days, range: 2–35 days) and hs-CRP (9.3 days, range: 2–20 days). The correlation of CT scanning and hs-CRP with the RT–PCR results has not been reported before. We found hs-CRP to have a shorter conversion time than other tests and may be used to predict the conversion of CT scanning, viral shedding and the prognosis of patients.

There were several limitations in this study. First, patients in this cohort were imported from various countries, so the variants might be a potential confounding factor on the viral shedding. Second, the detection window for each patient would be underestimated in this study due to the absence of precise records of the onset of symptoms, even though the relative differences of detection window between the three sample types were not influenced. Sample vacancy of some patients in the initial days brought bias to the sensitivity analyses, which provided underestimates in all the three sample types in this study. The study was limited to a small number of patients, and further longitudinal studies on a larger cohort would help to evaluate our conclusions. Based on our findings here, saliva was complementary to anal swab samples and can increase the reliability of discharge criteria of COVID-19 patients. “Double-check” with anal swab and saliva would decrease false negatives and help with the control of transmission from re-positive patients to the community.

## Author contributions

CQ contributed to conception and design, collected the specimens, acquisition of data, analysis and interpretation of data, design, drafting the article, accountable for all aspects of the work in ensuring that questions related to the accuracy or integrity of any part of the work are appropriately investigated and resolved. HL contributed to conception and design, interpretation of data, final approval of the version to be published, accountable for all aspects of the work in ensuring that questions related to the accuracy or integrity of any part of the work are appropriately investigated and resolved. JW contributed to analyse the data and drafting the article. ZS contributed to finish the laboratory test. SZ contributed to design the part of work. CT and TW contributed to collect the specimens. WL contributed to analyse the data. All authors read and approved the manuscript.
